# Improved RIDIT statistic approach provides more intuitive and informative interpretation of EQ-5D data

**DOI:** 10.1186/s12955-020-01313-3

**Published:** 2020-08-21

**Authors:** Abdelghafour Marfak, Ibtissam Youlyouz-Marfak, Youness El Achhab, Elmadani Saad, Chakib Nejjari, Abderraouf Hilali, Jack Turman Jr

**Affiliations:** 1 High Institute of Nursing Professions and Health Technics of Rabat, Rabat, Morocco; 2 Laboratory of Health Sciences and Technics, High Institute of Health Sciences, Hassan 1st University, Settat, Morocco; 3 Laboratory of Epidemiology, Clinical Research and Community Health, Faculty of Medicine and Pharmacy of Fez, Fes, Morocco; 4 Regional Centre for Careers Education and Training of Taza, Taza, Morocco; 5 Indiana University, Richard M. Fairbanks School of Public Health, Indianapolis, IN USA

**Keywords:** Improved RIDIT, Statistical analysis, EQ-5D-5 L, EQ-5D-3 L, Absolute risk reduction, Ordinal odds, Number needed to treat

## Abstract

**Background:**

EQ-5D is generic measure of health-related quality of life. Studies using EQ-5D generate ordinal data that are interpreted as categories ordered by severity. New analytic approaches taking into account the ordinal nature of the health dimension severity and leading to a better interpretation of EQ-5D data are needed to better elucidate differences in health-related quality of life. We propose utilizing the Improved RIDIT statistical method to analyze EQ-5D outcomes.

**Methods:**

556 Moroccan participants aged over 18 years representing four chronic diseases: back pain (*n* = 158), renal insufficiency (*n* = 56), diabetes (*n* = 82) or hypertension (*n* = 80) and healthy subjects (*n* = 180). All participants received the two EQ-5D versions. Two other published data sets were included. The first was extracted from a diabetic Spain study and the second was extracted from a clinical trial study. The Improved RIDIT analyses were carried out using an R statistic program we developed.

**Results:**

Applying the Improved RIDIT on the EQ-5D data allowed estimating for the first time the ordinal odds, the Absolute Risk Reduction (ARR) or the Absolute Risk Increase (ARI) and the Number Needed to Treat. The ARI values estimated for Moroccan patients showed that (i) hypertension increased anxiety/depression by 66% and reduced mobility by 65%; (ii) back pain increased pain/discomfort by 69%; (iii) renal insufficiency impacts mobility (ARI = 57%, odds_ordinal_ = 9.95) and usual activities (ARI = 44%, odds_ordinal_ = 6.41) and (iv) diabetes acts only on anxiety/depression (ARI = 50%, odds_ordinal_ = 4.8). Also, we demonstrated that the approach works well in clinical trials.

**Conclusions:**

Improved RIDIT provides more intuitive and informative interpretation of the EQ-5D data by (1) taking into account the level severity; estimating (2) the odds ordinal, (3) the ARR/ARI and the NNT; (4) analyzing the five dimensions of the EQ-5D separately, which gives clinical teams more precision in understanding the treatment/pathology impacts on the health status and completes the EQ-5D data analysis based on score utilities.

## Background

The EQ-5D instrument is a generic measure of patient reported outcomes. It was developed for estimating utility scores based on population preferences for the different health states. It is used in observational studies to evaluate health-related quality of life in patients with different pathologies [1–5], to estimate normative values of health states or so called score utilities in different populations [6–12] and in clinical trials to describe treatment efficacy [13, 14].

The EQ-5D exists in two versions called EQ-5D-3 L and EQ-5D-5 L and recently the child-friendly EQ-5D version (EQ-5D-Y) was introduced [15, 16]. The EQ-5D consists of five dimensions (Mobility, Self-care, Usual Activities, Pain/Discomfort and Anxiety/Depression). Each one has three (EQ-5D-3 L) or five (EQ-5D-5 L) levels of problems representing the degree of the health state severity. This descriptive system is a set of a number of health states, each coded by a number of 5 digits. The three levels version (EQ-5D-3 L) contains a set of 3^5^ = 243 health states, whereas the 5 L version contains 5^5^ = 3125 health states. To summarize the EQ-5D responses the individual response is converted into a utility score. Also, the EQ-5D consists of a Visual Analog Scale (VAS) to assess patients’ current health status (scale 0–100, where 0 = worse imaginable health state and 100 = best imaginable heath state). The VAS has been established as valid and reliable in a range of clinical and research applications [17, 18].

The EQ-5D instrument has been expressly developed to yield two forms of data: score utilities and VAS values. Appropriate analysis of EQ-5D data and consequent interpretation of results is of vital importance to the field. In studies whose objective is to compute quality-adjusted life years or in medical studies where the aim is to compare the quality of life between two groups of subjects (for example, healthy and ill; before and after receiving a treatment), the score utilities and the VAS values are most often used. When utility scores are missing, it would be acceptable to apply another country’s value set to estimate utilities. Utility scores and VAS are univariate continuous variables and the statistical inference can be done through the common Student or Mann-Whitney statistical tests. These statistics fail to reveal the difference between two samples of subjects at each dimension separately since the utilities and VAS measure the global health state by grouping the 5 dimensions in a single value. Measurement on the 5 distinct dimensions of the EQ-5D can also be informative in order to determine which dimensions of the EQ-5D are affected by disease or improved by treatment. Some studies [1, 2, 4, 14] have compared the impact of a treatment or disease on the quality of life by comparing the frequencies of “level 1” obtained from the two samples for each of the five dimensions. A chi-square test is often used to compare these frequencies. For example, in a clinical trial, the positive impact of treatment on the health status of patients is significant if the frequency of the “level 1” corresponding to “no problems” is observed to be higher after treatment [14]. Health-related quality of life studies using the EQ-5D generate outcome ordinal data that are interpreted as categories ordered by severity. Nevertheless, the Chi-square test fails to incorporate the ordered nature of the random variable even when used at each dimension separately. By dichotomizing the responses into two categories: “no problem: level 1” and “other problems: the remaining levels together”, the Chi-square masks the importance of the severity of the levels, which is the fundamental property of EQ-5D.

New analytic approaches taking into account the ordinal nature of the EQ-5D data are needed to better elucidate differences in health related quality of life. Bross developed a non-parametric test RIDIT (Relative to an Identified Distribution) to analyze ordered data [19]. The mean RIDIT comparison of two samples from two conditions estimates the probability that a randomly selected subject from one sample will have lower health state than a randomly selected subject from the other one. RIDIT analysis works well when the reference population is known, as this analysis can lead to conflicting conclusions depending on the group used as reference. In some cases notably when the sample sizes are very different, interchanging the groups can affect the standard deviation and the statistical result. To overcome this limitation, Flora proposed an “Improved RIDIT” approach [20] based on the Mann-Whitney test [21] and theoretical bases of Conover [22]. Contrary to Bross’ RIDIT analysis, the Improved RIDIT does not require a reference group. The main difference between RIDIT and Improved RIDIT relates to the probabilities under consideration. The Bross method considers only the probability that a randomly selected subject from a test group is in more serious condition than a randomly selected subject from the reference population group. The Improved RIDIT procedure considers three probabilities: more serious, as serious as and less serious condition.

To our knowledge, the Improved RIDIT has not yet been used to analyze EQ-5D data. The aim of our study is to apply the Improved RIDIT approach in the context of ordered data of health-related quality of life outcomes from EQ-5D. We demonstrate the efficacy and advantages of the Improved RIDIT analytical tool on 3 sets of EQ-5D data.

The improved RIDIT statistic method comes with an added value for the EQ-5D questionnaire by revealing information on health status changes for each EQ-5D dimension separately. Utility scores yield information on the change in overall health status. Together, utilities and improved RIDIT can provide total information on the change of health-related quality of life.

## Methods

### Data collection

We applied the Improved RIDIT method on three sets of EQ-5D data. The first data set (Set 1) was extracted from the Spanish study on diabetes by Mateo et al. [23]. The second data set (Set 2) was extracted from clinical trial study of Devlin et al. [14]. The third set (Set 3) were data collected in our study representing five Moroccan citizens groups: a sample of healthy subjects and 4 samples of chronic diseases (back pain, hypertension, renal insufficiency and diabetes).

We chose Set 1 because the authors used the EQ-5D-5 L version to evaluate the health-related quality of life of individuals with diabetes, a chronic disease that we incorporated in our data set (Set 3). We chose Set 2 for two reasons: (i) in their study, Devlin et al. [14] used the EQ-5D-3 L version, which is the case also in our study (Set 3); (ii) Devlin et al. evaluated in their clinical trial the positive impact of medical treatment on the quality of life of patients. Devlin et al. data set (Set 2) enabled us to test the effectiveness of the Improved RIDIT method to evaluate the health quality of life in clinical trials.

The 556 subjects associated with the Moroccan data (Set 3) were recruited from the unit of hypertensive treatment (Settat, Morocco), hemodialysis center (Temara, Morocco), Association de Soutien des Diabetiques de Settat (Settat, Morocco) and University Hospital of Rabat (Rabat, Morocco). All subjects had one of the four following diagnoses: back pain (*n* = 158), renal insufficiency (*n* = 56), diabetes (*n* = 82) or hypertension (*n* = 80). The control sample consisted of healthy subjects (*n* = 180) working in the clinical care setting. Only individuals who have none of the 4 pathologies studied are included in the healthy sample. All study participants were over 18 years of age and signed an informed consent form after receiving information on study objectives and the instrument used to collect data. For the back pain, hypertension and diabetes samples patients with hypertension/renal insufficiency/diabetes, back pain/renal insufficiency/diabetes and back pain/renal insufficiency/hypertension were excluded, respectively. For the renal insufficiency sample patients with hypertension or back pain were excluded. For the renal insufficiency group, 78 patients gave their consent from a total of 90 patients followed in the hemodialysis center. 56/78 met the exclusion criteria. For the diabetes and the hypertension samples, 82/135 and 80/127 patients gave their consent and met the exclusion criteria, respectively. For the control and back pain samples, 3 interviewers collected the data for two months (one day per week) with a frequency of 10 participants per interviewer per day. All participants were selected randomly with no repeated measurement. In total, we had the consent of *N* = 3 * 8 * 10 = 240 participants. For the back pain sample 158 patients met the exclusion criteria. For the control group 180 had no chronic pathology. The ethics committee of the Hassan 1st University (Settat, Morocco) provided approval of the study protocol.

Participants’ responses to the EQ-5D were collected according to the Scalone protocol [3]. Subjects from the clinical diagnostic groups completed the survey in the clinic setting; control subjects completed the survey at their place of employment. All subjects reported their age and gender and received the Arabic EQ-5D-5 L version followed by the Arabic 3 L version and finally the visual analogue scale (VAS) in accordance with previous research findings, which showed that when respondents scored the 3 L first, there could be a tendency to not use the “in-between” levels 2 and 4 of the 5 L [24]. All statistical analyses were carried out using an R statistic program we developed.

### Statistical analysis

#### The algorithm of the improved RIDIT method

Consider two randomly independent samples Y (healthy control or receiving a treatment in clinical trial) and X (patients with chronic disease or receiving a treatment of reference/placebo) from a study of health-related quality of life. The data obtained for each dimension of EQ-5D was summarized in a contingency table where rows refer to the Y and X samples and columns refer to the EQ-5D levels (3 and 5 columns for EQ-5D-3 L and EQ-5D-3 L, respectively) (Table 1). For each dimension of the EQ-5D data, the categorical random variable is ordered from the least serious level (“No Problem” is coded “1”) to the most serious level (“Extreme Problem” is coded “5” for the 5 L version).

**Table 1:** EQ-5D data format to calculate Improved RIDIT

Level of severity (***l***)	1	…	2	k	Total
**Sample Y**	${f}_Y^{(1)}$	…	${f}_Y^{\left(k-1\right)}$	${f}_Y^{(k)}$	**n**_**y**_
**Sample X**	${f}_X^{(1)}$	…	${f}_X^{\left(k-1\right)}$	${f}_X^{(k)}$	**n**_**x**_
**Total**	*t*_1_	…	*t*_*k* − 1_	*t*_*k*_	**N = n**_**y**_ **+ n**_**x**_

*k* is the number of the dimension levels, i.e., *k* = 3 and *k* = 5 for EQ-5D-3 L and EQ-5D-5 L, respectively. Levels L_1_, …, L_k_ are ordered from least to most severe. For each level $l=1,\dots, k,{f}_Y^{(l)}\;\mathrm{and}\;{f}_X^{(l)}$ denote the observed frequencies of the Y and X samples, respectively

Let y_j_ (j = 1, …, n_y_) denote the n_y_ observations of the reference sample (Y) and x_i_ (i = 1, …, n_x_) denote the n_x_ observations of the comparison sample (X).

Define the function ω as:

1 $$ {\omega}_{ij}=\left\{\begin{array}{c}+1\kern0.75em if\ {x}_i>{y}_j\ \\ {}\kern0.75em 0\kern1em if\ {x}_i={y}_j\ \\ {}-1\kern0.75em if\ {x}_i<{y}_j\ \end{array}\right. $$

From the function ω, we define the following probabilities:

2 $$ {\pi}^{+}=P\left[X>Y\right] $$

3 $$ {\pi}^0=P\left[X=Y\right] $$

4 $$ {\pi}^{-}=P\left[X<Y\right] $$

π^+^, π^0^ and π^−^ indicate that subjects randomly selected from the X sample are in a worse, in a same or in a better health state, respectively than subjects randomly selected from the Y sample.

The Improved RIDIT test is based on the statistic:

5 $$ W=\sum \limits_{i=1}^{n_x}\sum \limits_{j=1}^{n_y}{\omega}_{ij} $$

With

6 $$ E\left[W\right]=\sum \limits_{i=1}^{n_x}\sum \limits_{j=1}^{n_y}E\left[{\omega}_{ij}\right] $$

From Eqs. 1, 2, 3 and 4, we obtain:

7 $$ E\left[W\right]=\sum \limits_{i=1}^{n_x}\sum \limits_{j=1}^{n_y}\left({\pi}^{+}-{\pi}^{-}\right)\kern0.5em $$

8 $$ \mathrm{So},E\left[W\right]={n}_y{n}_x\left({\pi}^{+}-{\pi}^{-}\right) $$

Then an estimate of (*π*^+^ − *π*^−^) is:

9 $$ {\hat{\pi}}^{+}-{\hat{\pi}}^{-}=\frac{W}{n_y{n}_x} $$

From (Eq. 1) and Table 1, we re-write (Eq. 5) as:

10 $$ W=\sum \limits_{l=1}^{k-1}\left({f}_Y^{(l)}\sum \limits_{p=l+1}^k{f}_X^{(p)}\right)-\sum \limits_{l=2}^k\left({f}_Y^{(l)}\sum \limits_{p=1}^{l-1}{f}_X^{(p)}\ \right)\kern0.5em $$

The variance of W was given by Flora [20]:

11 $$ Var(W)=\frac{n_y{n}_x\left(N+1\right)}{3}\left(1-\frac{\sum \limits_{l=1}^k\left({t}_l^3-{t}_l\right)}{N^3-N}\right)\kern0.5em $$

From (eq.3), an estimate of π^0^ is:

12 $$ {\hat{\pi}}^0=\frac{1}{n_y{n}_x}\sum \limits_{l=1}^k{f}_Y^{(l)}{f}_X^{\left(\mathrm{l}\right)}\kern0.5em $$

From the probability propriety: *π*^0^ + *π*^−^ + *π*^+^ = 1 and (Eq. 9), estimates of π^+^ and π^−^ are respectively:

13 $$ {\hat{\pi}}^{+}=\frac{1}{2}\left(1-{\hat{\pi}}^0+\frac{W}{n_y{n}_x}\right)\kern0.5em $$

14 $$ {\hat{\pi}}^{-}=\frac{1}{2}\left(1-{\hat{\pi}}^0-\frac{W}{n_y{n}_x}\right)\kern0.5em $$

Therefore, estimation of *π*^−^ and *π*^+^ requires calculation of W from Eq. 10, which is not trivial. However, we propose the following simple algorithm:

1. Transform the contingency table (Table 1) into a square matrix ***ω***_(***k***, ***k***)_ whose elements are the products of the frequencies ${f}_X^{(l)}$ and ${f}_Y^{(m)}$ (*l, m = 1, …, k*) and *k* is the level number of the ordinal variable.
  $$ \boldsymbol{\omega} =\left(\begin{array}{ccccc}{f}_Y^{(1)}{f}_X^{(1)}& {f}_Y^{(1)}{f}_X^{(2)}& \cdots & {f}_Y^{(1)}{f}_X^{\left(k-1\right)}& {f}_Y^{(1)}{f}_X^{(k)}\\ {}{f}_Y^{(2)}{f}_X^{(1)}& {f}_Y^{(2)}{f}_X^{(2)}& \cdots & {f}_Y^{(2)}{f}_X^{\left(k-1\right)}& {f}_Y^{(2)}{f}_X^{(k)}\\ {}\vdots & \vdots & \ddots & \vdots & \vdots \\ {}{f}_Y^{\left(k-1\right)}{f}_X^{(1)}& {f}_Y^{\left(k-1\right)}{f}_X^{(2)}& \cdots & {f}_Y^{\left(k-1\right)}{f}_X^{\left(k-1\right)}& {f}_Y^{\left(k-1\right)}{f}_X^{(k)}\\ {}{f}_Y^{(k)}{f}_X^{(1)}& {f}_Y^{(k)}{f}_X^{(2)}& \cdots & {f}_Y^{(k)}{f}_X^{\left(k-1\right)}& {f}_Y^{(k)}{f}_X^{(k)}\end{array}\right) $$

Looking at the ***ω***_(***k***, ***k***)_ matrix, we notice that:

- The sum of all entries in ***ω***_(***k***, ***k***)_ is equal to *n*_*x*_*n*_*y*_.
- The sum of all entries above the main diagonal is equivalent to the left term in (eq. 10). These values ${f}_Y^{(m)}\ {f}_X^{(l)}\kern0.50em \left(l>m\right)$ indicate that a randomly subject selected from X is in a high level of the ordinal variable (worse heath state in the EQ-5D dimension) than a randomly subject selected from Y.
- The sum of all entries below the main diagonal is equivalent to the right term in eq. 10. These entries ${f}_Y^{(m)}\ {f}_X^{(l)}\kern0.50em \left(l<m\right)$ indicate that a randomly selected subject from X is in a low level of the ordinal variable (better heath state in the EQ-5D dimension) than a randomly selected subject from Y.
- The sum of the diagonal elements (the trace of the ***ω***_(***k***, ***k***)_ matrix) divided by *n*_*x*_*n*_*y*_ is equivalent to (Eq. 12).

2. Estimate *π*^0^ by ${\hat{\pi}}^0= Tr\left(\boldsymbol{\omega} \right)/{n}_x{n}_y$ where *Tr* is the trace.
3. Construct the ***W***_(***k***, ***k***)_ matrix by multiplying the diagonal elements of the ***ω***_(***k***, ***k***)_ matrix by zero (*x*_*i*_ = *y*_*j*_ from Eq. 1), the elements above the diagonal of ***ω***_(***k***, ***k***)_ by 1 (*x*_*i*_ > *y*_*j*_ from Eq. 1) and the elements below the diagonal of ***ω***_(***k***, ***k***)_ by − 1 (*x*_*i*_ < *y*_*j*_ from Eq. 1):
  $$ \boldsymbol{W}=\left(\begin{array}{ccccc}0& {f}_Y^{(1)}{f}_X^{(2)}& \cdots & {f}_Y^{(1)}{f}_X^{\left(k-1\right)}& {f}_Y^{(1)}{f}_X^{(k)}\\ {}-{f}_Y^{(2)}{f}_X^{(1)}& 0& \cdots & {f}_Y^{(2)}{f}_X^{\left(k-1\right)}& {f}_Y^{(2)}{f}_X^{(k)}\\ {}\vdots & \vdots & \ddots & \vdots & \vdots \\ {}-{f}_Y^{\left(k-1\right)}{f}_X^{(1)}& -{f}_Y^{\left(k-1\right)}{f}_X^{(2)}& \cdots & 0& {f}_Y^{\left(k-1\right)}{f}_X^{(k)}\\ {}-{f}_Y^{(k)}{f}_X^{(1)}& -{f}_Y^{(k)}{f}_X^{(2)}& \cdots & -{f}_Y^{(k)}{f}_X^{\left(k-1\right)}& 0\end{array}\right) $$
4. Calculate W (Eq. 10) as the sum of all the entries of the ***W***_(***k***, ***k***)_ matrix.
5. Estimate ${\hat{\pi}}^{+}$ as the sum of all the entries above the diagonal of the ***W***_(***k***, ***k***)_ matrix divided by *n*_*x*_*n*_*y*_.
6. Estimate ${\hat{\pi}}^{-}$ as the absolute value of the sum of all the entries below the diagonal of the ***W***_(***k***, ***k***)_ matrix divided by *n*_*x*_*n*_*y*_.

Notice that ${\hat{\pi}}^{-},{\hat{\pi}}^{+},{\hat{\pi}}^0$ and W estimated in the steps 2 and 4–6 verify the Eqs (13) and (14).

#### The improved RIDIT statistic

Once the quantities π^+^ and π^−^ are estimated, the Improved RIDIT method leads to study the inference between two randomly samples X and Y under two experimental conditions by three statistics:

##### The standard distribution test

If the two samples Y and X came from the same population so the null hypothesis H0: *π*^+^ = *π*^−^. Consequently, the expected value of W is zero. Alternatively, if X and Y come from two different populations then the alternative hypothesis H1: *π*^+^ ≠ *π*^−^. For example, let X be a sample of persons suffering of back pain and Y a sample of healthy persons and consider the dimension pain/discomfort in EQ-5D. Testing the hypothesis that these two populations are equivalent with respect to pain/discomfort severity is equivalent to testing the null hypothesis *H*0 (*π*^+^ = *π*^−^) against the alternative one *H*1 (*π*^+^ ≠ *π*^−^). *H1* indicates that persons with back pain will be more seriously in a state of pain/discomfort than healthy persons. Conover [22] demonstrated that for large samples X and Y (sample size greater than 30, which is a general rule of thumb for the large sample condition), W is approximately normally distributed. Therefore, the test statistic for the hypothesis that the two populations are the same regarding the level of severity is constructed as the following:

15 $$ Z=\frac{W}{\sqrt{Var(W)}} $$

The null hypothesis is rejected in favor to the alternative one if the Z value is greater than the threshold value of the standard distribution at the significance level α. In the context of EQ-5D data, the Improved RIDIT performing the simultaneous comparability of the five EQ-5D dimensions. However, these simultaneous tests are not independent since they share the same data. In this case of multiple testing, a common strategy is to adjust the significance level as a function of the number of tests we are running. In order to reduce the possibility of getting false positives, we used the Bonferroni-Holm procedure [25] to adjusting the *p*-values calculated by the Improved RIDIT.

##### The absolute risk reduction (ARR) and needed number to treat (NNT)

Considering the case of a study of the impact of treatment on health-related quality of life where X and Y are two randomly independent samples representing Control/or placebo and Treatment groups, respectively. If the treatment has a positive impact on the quality of life, then π^−^ = P(Y < X) indicates the probability that a random individual from the Treatment group has a better outcome than a random individual from the Control/placebo group and π^+^ = P(Y > X) indicates the probability that a random subject from the Control/placebo group has a preferable outcome than a random individual from the Treatment group. For the ordinal variable, Shepstone [26] showed that P(Y < X) - P(Y > X) is equivalent to the Absolute Risk Reduction (ARR). Walters estimated the ARR in the case of ordinal data by the use of the MWW U statistics. Walters proposed *ARR* = (*U*_*YX*_ − *U*_*XY*_)/*n*_*x*_*n*_*y*_ where U_YX_ and U_XY_ are the U statistics and *n*_*x*_ and *n*_*y*_ are the sample sizes of X and Y [27]. Walters discussed the limitation of this estimation. If there are no ties in the data (*π*^*0*^ = P(X = Y) = 0) then the ARR can be estimated exactly. But in the presence of a large amount of ties (*π*^*0*^ > 0) in the data, which is the case of EQ-5D outcomes, the ARR using the MWW approach can only be estimated approximately. Hence, approaches leading to exact estimation of P(Y < X) and P(Y > X) (π^−^ and π^+^, respectively) are welcome. Applying the Improved RIDIT to the health-related data, the ARR can easily be estimated by:

16 $$ \hat{ARR}={\hat{\pi}}^{-}-{\hat{\pi}}^{+} $$

Also, applying the Improved RIDIT to health-related quality of life data allows estimating the Needed Number to Treat (NNT). In clinical trials it is very important to estimate the NNT to evaluate the efficacy of a treatment. The NNT is defined as the number of patients who would have to receive a treatment to prevent one additional bad outcome [28]. It is estimated by NNT = 100/ARR (where ARR is %) or NNT = 1/ARR (where ARR is proportion).

In the case of studies concerning the evaluation of the negative effect of pathologies on the health-related quality of life, we can define the Absolute Risk Increase (ARI) similarly to the ARR in the clinical trials studies. The ARI is estimated by:

17 $$ \hat{ARI}={\hat{\pi}}^{+}-{\hat{\pi}}^{-} $$

where π^+^ is the probability that a randomly patient from the disease group has a worse outcome than a randomly selected healthy subject from the control group.

Both ARR and ARI are positive estimates of the impact of a treatment or a disease on the quality of life. The confidence interval for the ARR/ARI at the confidence level (1-α) is done by:

18 $$ {CI}_{1-\frac{\alpha }{2}}=\frac{\left|W\right|}{n_y{n}_x}\pm \frac{Z_{\frac{\alpha }{2}}}{n_y{n}_x}\sqrt{Var(W)} $$

The impact of the treatment/disease is statistically significant if the confidence interval of ARR/ARI does not include zero.

##### The odds ordinal

Agresti [29] has proposed a generalization of the odds to two independent ordinal variables X and Y as:

19 $$ {odds}_{ordinal}=\frac{P\left(Y>X\right)}{P\left(X>Y\right)} $$

Applying the Improved RIDIT to the EQ-5D data obtained from two randomly independent samples X and Y, we can easily estimate the ordinal odds by:

20 $$ {\hat{odds}}_{ordinal}=\frac{{\hat{\pi}}^{+}}{{\hat{\pi}}^{-}} $$

Considering one of the five dimensions of the EQ-5D, odds_ordinal_ means that patients are odds_ordinal_ times in worse health dimension than the healthy ones.

Similarly, for clinical trials if X and Y are two independent samples of subjects receiving randomly a reference treatment/placebo and a treatment of interest, respectively the odds is estimated by:

21 $$ {\hat{odds}}_{ordinal}=\frac{{\hat{\pi}}^{-}}{{\hat{\pi}}^{+}} $$

which means that patients are odds_ordinal_ times in better health dimension after receiving treatment.

#### Simulation

##### EQ-5D data generation

**Fig. 1:**
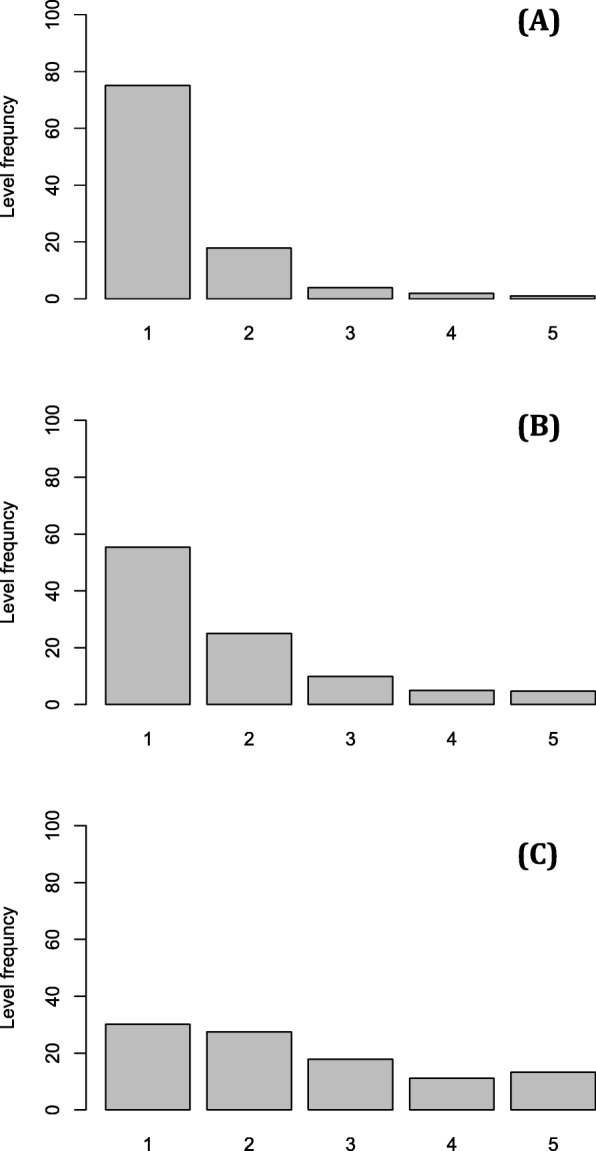
Distributions of the five levels of an EQ-5D-5 L dimension simulated from an exponential density of a rate λ as described in the method section. λ = 1.4 (A), λ = 1.0 (B) and λ = 0.6 (C)

To generate EQ-5D outcomes that are likely to occur in practice, we used the exponential distribution f(x) = λe^−λx^. Different values of the rate λ (Fig. 1) were used to generate different shapes of frequencies for the two EQ-5D-5 L and EQ-5D-3 L versions according to the following procedure:

- For each value of λ, 10,000 observations have been generated.
- We replaced each observation by its integer value plus one.
- For the simulation of 5 L data, if an observation is greater than 5 it is replaced by 5. For 3 L data, if an observation is greater than 3 it is replaced by 3.

Figure 1 shows the decrease of the level “1” (no problems) frequency (from Fig. 1a to Fig. 1c) and the increase of the other level frequencies. This illustrates typically what could be occurring in patient outcomes when the disease affects the health-related quality of life. Interpreting Fig. 1 in the strand sense (from Fig. 1c to Fig. 1a), we observe improvement of the patient outcomes (increasing of the “no problem” level and decreasing of the other levels). This indicates a positive effect of a medical treatment on the quality of life.

#### Comparison between the improved RIDIT and Mann-Whitney-Wilcoxon (MWW)

To compare the two methods, the computer simulation was based on the comparison between the false positive rate and the power estimated by each of the two approaches. This algorithm simulation was based on the analysis of false positive rate and power estimation.

##### False positive rate estimation

1. For each of the three profiles (Fig. 1a, b and c), draw with replacement two independent random samples X and Y of size n_x_ and n_y_, respectively. The sample sizes used were 30, 50, 100, 200 and 300. In theses simulations, we consider situations where the distributions have the same shape (the null hypothesis is true).
2. The test statistic, Improved RIDIT or MWW, was calculated between the X and Y samples. If the calculated test is lower than the critical value of the test statistic for a nominal *α*, a false positive was recorded. This step was carried out for all the combinations (n_x_, n_y_) sizes.
3. Steps 1 and 2 were repeated S times. We estimated the false positive rate ($\hat{FP}\Big)$ by the proportion of false positives among the S repetitions (in all simulations, S = 10,000).

##### Power estimation

Consider situations where the two distributions to be compared do not have the same shape. For this, suppose the distribution profile in Fig. 1a representing a control/treatment group (sample X) and the profiles in Fig. 1b and c representing a disease group (sample Y).

1. Draw two independent random samples X and Y (X from Fig. 1a and y from Fig. 1b or Fig. 1c) of size *n*_*x*_ (30, 50, 100, 200 or 300) and *n*_*y*_ (30, 50, 100, 200 or 300), respectively.
2. For each combination (n_x_, n_y_), the Improved RIDIT and MWW tests were calculated. If the test value is greater or equal than the critical value of the test statistic for a nominal α, a success is recorded (the alternative hypothesis is true).
3. Steps 1 and 2 were repeated S times. We estimated the power ($\hat{P}\Big)$ by the proportion of successes among the S repetitions (in all simulations, S = 10,000).

#### On the use of the odds ordinal and the ARR/ARI

The ARR/ARI and the odds_ordinal_ are connected by the relationship:

${odds}_{ordinal}=1+\frac{ARR}{\pi^{-}}$, in the case of comparing a treatment group to a control group.

${odds}_{ordinal}=1+\frac{ARI}{\pi^{+}}$, in the case of comparing a healthy group to a control group.

To analyze deeply this relationship, we simulated values of π^−^ and π^+^ for fixed values of ARR/ARI. At first the ARR/ARI values were generated according to a normal distribution. At a second time, at each simulation 10, 000 values of π^−^ and π^+^ were generated from a uniform distribution. After, we retained only the values π^−^ and π^+^ whose difference was equal to the ARR/ARI values generated in the first step.

## Results

### Simulation results

#### Simulation result of the comparison between MWW and improved RIDIT

Table 2 summarizes comparisons of the false positive rates obtained by Improved RIDIT and MWW over the three selected distributions depicted in Fig. 1 for each of the 25 sample size combinations. These results demonstrated the robustness of the Improved RIDIT and MWW tests since all the false positive rate values obtained by the two methods did not greatly exceed the nominal level of significance (α = 0.05) (Table 2). In addition, for the three distributions and all the sample sizes examined, the Improved RIDIT always estimated less false positives than MWW. In contrast, MWW some times gave falsely significant results, i.e., the false positive rate exceeded 5% (Table 2).

**Table 2:** False positive rate detected by the Improved RIDIT and MWW relative to nominal *α* = 0.05 using simulation under the null hypothesis. Three examples of distributions under H0 were used according to the level frequency profiles in Fig. 1

Sample sizes	H0 profile, Fig. 1a		H0 profile, Fig. 1b		H0 profile, Fig. 1c	
	Improved RIDIT	MWW	Improved RIDIT	MWW	Improved RIDIT	MWW
**30, 30**	0.0482	0.0511	0.0498	0.0505	0.0502	0.0523
**30, 50**	0.0428	0.0454	0.0491	0.0516	0.0458	0.0468
**30, 100**	0.0497	0.0519	0.0462	0.0482	0.0452	0.0478
**30, 200**	0.0445	0.0473	0.0475	0.0497	0.0482	0.0504
**30, 300**	0.0452	0.0474	0.0478	0.0499	0.0454	0.0474
**50, 30**	0.0476	0.0483	0.0486	0.0490	0.0431	0.0446
**50, 50**	0.0468	0.0486	0.0489	0.0509	0.0491	0.0541
**50, 100**	0.0477	0.048	0.0494	0.0534	0.0454	0.0470
**50, 200**	0.0442	0.0463	0.0492	0.0509	0.0498	0.0542
**50, 300**	0.0478	0.0495	0.0464	0.0483	0.0476	0.0492
**100, 30**	0.0483	0.0503	0.0481	0.0501	0.0454	0.0471
**100, 50**	0.0451	0.0463	0.0428	0.0446	0.0496	0.0504
**100, 100**	0.0449	0.0467	0.0451	0.0474	0.0482	0.0505
**100, 200**	0.0479	0.0499	0.0474	0.0498	0.0478	0.0495
**100, 300**	0.0501	0.0543	0.0455	0.0468	0.0492	0.0535
**200, 30**	0.0496	0.0503	0.0486	0.0490	0.0496	0.0501
**200, 50**	0.0421	0.0441	0.0494	0.0529	0.0473	0.0485
**200, 100**	0.0487	0.0500	0.0467	0.0489	0.0462	0.0487
**200, 200**	0.0462	0.0485	0.0469	0.0489	0.0483	0.0501
**200, 300**	0.0494	0.0526	0.0475	0.0489	0.0499	0.0510
**300, 30**	0.0451	0.0475	0.0461	0.0483	0.0482	0.0510
**300, 50**	0.0467	0.0489	0.0496	0.0505	0.0492	0.0509
**300, 100**	0.0492	0.0530	0.0471	0.0494	0.0497	0.0483
**300, 200**	0.0487	0.0495	0.0475	0.0487	0.0491	0.0505
**300, 300**	0.0497	0.0511	0.0462	0.0475	0.0494	0.0509

**Fig. 2:**
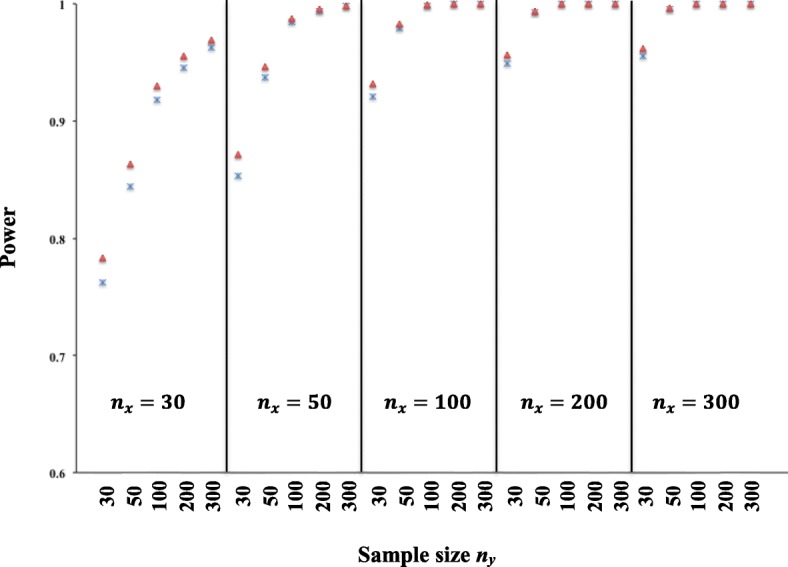
Power estimated by MWW (Δ) and Improved RIDIT (×) for comparison between two randomly independent samples X (size, nx) and Y (size, ny) simulated from the EQ-5D dimension profiles in Fig. 1a and Fig. 1c, respectively. Simulation was based on 10, 000 replications and a nominal α = 0.05 (two-sided)

Investigation of the power was based on the comparison between two simulated EQ-5D dimension distributions like as those presented in Fig. 1. We simulated two randomly independent samples X and Y whose distributions were similar to Fig. 1a and Fig. 1c, respectively. Analysis of the power values estimated by MWW and Improved RIDIT (Fig. 2) demonstrated that the power increases with the sample size. The power of the two tests was greater than 0.8 except in the case where the two samples had a size equal to 30. If there are fewer subjects in one of the two samples (size < 50), we observed a small difference between the power values of the MMW and improved RIDIT tests. This difference vanished for sample sizes greater than 50.

#### Simulation result on the use of the ordinal odds and ARR/ARI

**Fig. 3:**
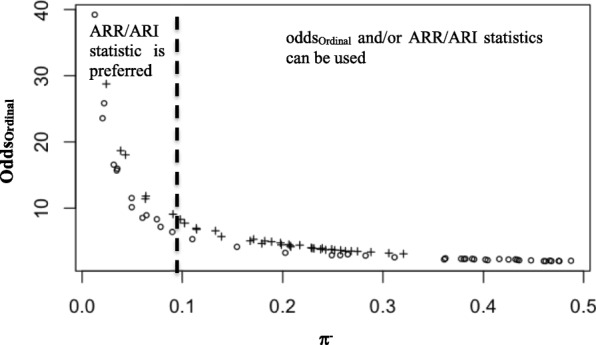
Simulation results of oddsOrdinal versus π-. Odds were simulated for two values of ARR (0.5 (o) and 0.7 (+)) at different values of π-. ARR were simulated from normal distributions with standard deviation = 0.02 and mean = 0.5 and mean = 0.7. π- values were simulated from a uniform distribution

Figure 3 shows odds_ordinal_ versus π^−^ obtained from two simulations corresponding to ARR = 0.5 and ARR = 0.7. We observed that the lower the risk in the control group (π^−^), the larger the difference between odds_ordinal_ and ARR. Also, the odds_ordinal_ overestimated the risk when π^−^ is too low (< 10%).

### Data set 1

We applied the Improved RIDIT statistics (Table 3) on EQ-5D-5 L data used to evaluate 1857 individuals (1016 women and 841 men) with diabetes living in Spain [23]. The study results revealed that diabetes affects the health related quality of life outcomes of subjects, affecting the health of women more than that of man by acting on all five dimensions of health. Findings from this study were based on comparisons between two frequencies f1 (obtained for “level 1”) and f2 (obtained as the sum of the four others), which masks information on the severity of each level. By applying the Improved RIDIT method on the same data set, the likelihood that diabetic women are in a worse state than diabetic men was estimated by three statistics (Table 3): the standard statistic test (W-test), The ARI and the ordinal odds. In term of risk difference, the ARI values showed that diabetic women have an absolute risk increase of 22% in mobility, 16% in self care, 23% in usual activities, 29% in pain discomfort and 20% in anxiety depression than men. The odds ordinal values showed similar result demonstrating that diabetes affect the mobility, self care, usual activities, pain discomfort and anxiety for about 2 times in women than in men (Table 3). Also, the difference between diabetic women and men on health-related quality was demonstrated by the W-test. Indeed, the Bonferroni-Holm adjusted *p*-values calculated for the five dimensions (Table 3) were highly significant.

**Table 3:** Comparison between diabetic women and men on health-related quality of life by applying the Improved RIDIT analysis to data from Mateo et al. [23]

EQ-5D dimension	ARI [CI_**min**_ – CI_**max**_]	Odds_**Ordina**l_ [CI_**min**_ – CI_**max**_]	W-test (***P***_***HB***_***-value***)
**Mobility**	0.22 [0.17–0.27]	2.00 [1.76–2.28]	8.88 (< 0.0001)
**Self Care**	0.16 [0.12–0.20]	2.33 [2.07–2.73]	8.12 (< 0.0001)
**Usual Activities**	0.23 [0.19–0.28]	2.35 [2.07–2.67]	9.90 (< 0.0001)
**Pain/Discomfort**	0.29 [0.24–0.34]	2.38 [2.10–2.71]	11.35 (< 0.0001)
**Anxiety/Depression**	0.20 [0.15–0.24]	2.54 [2.15–2.80]	9.07 (< 0.0001)

*P*_*HB*_*-value* the Bonferroni-Holm *p-values* correction

### Data set 2

We also tested the Improved RIDIT method on the health-related quality of life data obtained through the EQ-5D-3 L version administered by Devlin et al. [14]. In contrast to Mateo et al. [23] analyzing the effect of pathology on the quality of life of patients, Devlin et al. evaluated the impact of a treatment (Enzalutamide) on the quality of life of patients aged ≥ 18 with castration-resistant prostate cancer. In total, 1717 patients were randomized to receive Enzalutamide (*n* = 872) or placebo (*n* = 845) [14].

Using the chi-square test, they showed that the effect of enzalutamide on health-related quality of life was significant in the pain/discomfort and anxiety/depression dimensions at week 13. Also, they find that enzalutamide improved the usual activities and pain/discomfort at week 25. There were no significant between-group differences in the mobility and self-care dimensions (Table 4). All these analyses were based on comparison of the percentages of level 1 “no problems” to the other levels, which does not take into account the ordinal nature of the data. Applying the Improved RIDIT method on Devlin’s data and using the Bonferroni-Holm adjusting for multiple testing (Table 4), we found that pain/discomfort (*P*_*BH*_-value = 0.007) and anxiety/depression (*P*_*BH*_-value = 0.013) dimensions were improved at week 13. At week 25, a difference was observed in pain/discomfort after adjusting *p*-values (*P*_*BH*_-value = 0.033) but not for usual activities (*P*_*BH*_-value = 0.124) (Table 4). The ARR statistics showed that enzalutamide decreased significantly the pain/discomfort by 8,9% and by 9,1% at week 13 and week 25, respectively. Also, the treatment reduced the anxiety/depression by 7,5% at week 13. The usual activities dimension was in the limit of signification at week 13 (*p-*value = 0.058), which was confirmed by the Bonferroni-Holm adjusting (*P*_*BH*_-value = 0.175). Enzalutamide, however, increased the usual activities at week 25 by ARR = 6,2% but not significantly due to the adjusted *p*-value (*P*_*BH*_-value = 0.124). In terms of odds ordinal ratio, the positive effect of enzalutamide on pain/discomfort was estimated by 1.42 times and 1.44 times at week 13 and week 25, respectively (Table 4). At week 13, the patients receiving enzalutamide were 1.43 times less anxious.

**Table 4:** Comparison between patients receiving Enzalutamid and those receiving placebo on health-related quality of life by applying the Improved RIDIT analysis to data from Devlin et al. [14]

Placebo vs Enzalutamid	Improved RIDIT						Chi-square
Week 13	${\hat{\pi}}^{-}$	${\hat{\pi}}^0$	${\hat{\pi}}^{+}$	ARR [95% CI]	Odds ordinal [95% CI]	*P*_*BH*_*-*value	*P*_*BH*_*-*value (a)
Mobility	0.218	0.602	0.181	0.036 [− 0.011; 0.082]	1.12 [0.98; 1.47]	0.254	0.319 (0.319)
Self Care	0.075	0.868	0.057	0.018 [−0.009; 0.045]	1.31 [0.94; 1.83]	0.167	0.450 (0.225)
Usual Activities	0.227	0.592	0.180	0.047 [0.000; 0.094]	1.26 [1.04; 1.53]	0.175	0.204 (0.068)
Pain/Discomfort	0.301	0.487	0.212	0.089 [0.037; 0.142]	1.42 [1.21; 1.67]	0.007	0.025 (0.005)
Anxiety/Depression	0.248	0.579	0.173	0.075 [0.027; 0.122]	1.43 [1.19; 1.71]	0.013	0.024 (0.006)
**Week 25**							
Mobility	0.215	0.597	0.188	0.027 [−0.029; 0.082]	1.14 [0.89; 1.47]	0.257	0.582 (0.582)
Self Care	0.092	0.850	0.058	0.034 [0.001; 0.067]	1.58 [1.11; 2.26]	0.157	0.309 (0.103)
Usual Activities	0.223	0.616	0.161	0.062 [0.008; 0.116]	1.39 [1.10; 1.75]	0.124	0.104 (0.026)
Pain/Discomfort	0.296	0.498	0.206	0.091 [0.029; 0.153]	1.44 [1.19; 1.75]	0.033	0.080 (0.016)
Anxiety/Depression	0.223	0.607	0.170	0.053 [−0.002; 0.108]	1.31 [1.04; 1.66]	0.132	0.322 (0.161)

CI Confidence Interval, ^**a**^*P-*values calculated in [14] using the chi-square test to compare between the placebo and Enzalutamid groups basing on the percentage of patients with (2 or 3) or without (1) problems in each EQ-5D dimension. *P*_*BH*_*-*value the Bonferroni-Holm *p-*values correction

### Data set 3

Table 5 shows the characteristics of the study participants. For the 5 samples, females comprise 55–60% of participants in all groups other than hypertension for which 86% of the group is female. The mean age was 36 years (SD = 10 years) for the control group, 38 years (SD = 10 years) for back pain, 52 years (SD = 16 years) for diabetic patients, 61 years (SD = 11 years) for hypertensive patients and 55 years (SD = 17 years) for people with renal insufficiency. Patients with chronic hypertension or renal insufficiency were the oldest with 58.7 and 51.8% with an age greater than 60 years. The response frequencies across 3 and 5 levels of EQ-5D dimensions are presented in Table S1 (Supplementary Material).

**Table 5:** Characteristics of the study samples

		Condition				
		Control	Back Pain	Diabetes	Hypertension	Renal Insufficiency
**Age (years)**	[18–40[	97 (53.9%)	79 (50.0%)	15 (18.3%)	3 (3.8%)	12 (21.4%)
	[40–60[	72 (40.0%)	63 (39.9%)	36 (43.9%)	30 (37.5%)	15 (26.8%)
	≥ 60	11 (6.1%)	16 (10.1%)	31 (37.8%)	47 (58.7%)	29 (51.8%)
**Sex**	Female	107 (59.4%)	95 (60.1%)	45 (54.9%)	69 (86.2%)	32 (57.1%)
	Male	73 (40.6%)	63 (39.9%)	37 (45.1%)	11 (13.8%)	24 (42.9%)

Analyses of the 5 L questionnaire data by the Improved RIDIT statistic are presented in Table 6. Considering the control sample as a reference, the absolute risk increase and the ordinal odds values for each disease and each health dimension were estimated. Also, the W-test and the associated Bonferroni-Holm *p*-values corrections were calculated (Table 6). The ARI values show that hypertension reduced mobility by 65%, self-care by 34% and usual activities by 32%. This pathology increased pain/discomfort by 49% and anxiety/depression by 66%. Regarding the ordinal odds values, hypertensive patients were 12.71 times less mobile, 14.42 times less autonomous and 4.24 times less active than healthy individuals. They suffer from pain 4 times and anxiety 8 times higher than the healthy ones. These findings were consolidated by the W-test where all the Bonferroni-Holm adjusted values were much smaller than the significance level.

**Table 6:** Improved RIDIT analysis of EQ-5D-5 L health-related quality of life outcomes of healthy subjects (reference group) versus sick subjects

	EQ-5D dimension	ARI [CI_**min**_ – CI_**max**_]	Odds_**Ordina**l_ [CI_**min**_ – CI_**max**_]	W-test (***P***_***BH***_***-***value)
**Hypertension**	**Mobility**	0.65 [0.52–0.77]	12.71 [9.35–17.29]	9.93 (< 0.0001)
	**Self Care**	0.34 [0.25–0.44]	14.42 [9.71–21.40]	7.27 (< 0.0001)
	**Usual Activities**	0.32 [0.20–0.44]	4.24 [2.90–6.20]	5.40 (< 0.0001)
	**Pain/Discomfort**	0.49 [0.35–0.63]	4.75 [3.22–7.00]	6.74 (< 0.0001)
	**Anxiety/Depression**	0.66 [0.52–0.80]	8.26 [5.59–12.21]	9.09 (< 0.0001)
**Back Pain**	**Mobility**	0.27 [0.17–0.37]	3.69 [2.75–4.95]	5.49 (< 0.0001)
	**Self Care**	0.27 [0.19–0.35]	10.48 [7.61–14.44]	6.70 (< 0.0001)
	**Usual Activities**	0.32 [0.22–0.42]	4.23 [3.14–5.69]	6.24 (< 0.0001)
	**Pain/Discomfort**	0.69 [0.58–0.81]	12.11 [9.04–16.21]	11.53 (< 0.0001)
	**Anxiety/Depression**	0.36 [0.24–0.47]	3.23 [2.41–4.32]	6.12 (< 0.0001)
**Diabetes**	**Mobility**	0.22 [0.11–0.32]	3.10 [2.05–4.69]	3.96 (0.0006)
	**Self Care**	0.05 [−0.01–0.1]	2.26 [1.05–4.89]	1.53 (0.247)
	**Usual Activities**	0.06 [−0.04–0.16]	1.48 [0.86–2.54]	1.23 (0.187)
	**Pain/Discomfort**	0.11 [− 0.02–0.25]	1.43 [0.86–2.38]	1.63 (0.318)
	**Anxiety/Depression**	0.50 [0.36–0.64]	4.80 [3.25–7.09]	6.99 (< 0.0001)
**Renal Insufficiency**	**Mobility**	0.57 [0.44–0.71]	9.95 [6.86–14.43]	8.27 (< 0.0001)
	**Self Care**	0.35 [0.25–0.44]	14.78 [9.28–23.54]	6.96 (< 0.0001)
	**Usual Activities**	0.44 [0.31–0.58]	6.41 [4.25–9.68]	6.54 (< 0.0001)
	**Pain/Discomfort**	0.37 [0.21–0.53]	3.12 [1.96–4.98]	4.58 (< 0.0001)
	**Anxiety/Depression**	0.27 [0.12–0.43]	2.45 [1.56–3.86]	3.44 (< 0.001)

*P*_*BH*_ value the Bonferroni-Holm *p-*values correction

For individuals with back pain, the adjusted *p*-values for multiple testing demonstrated that the health-related quality of life was significantly affected in patients (Table 6). The most affected dimension was pain/discomfort with an absolute risk increase of 69% and ordinal odds of 12.11. The four other dimensions were also affected from 27 to 36% (Table 6).

Renal insufficiency impacts the quality of life of patients by acting on mobility (ARI = 57%, odds_ordinal_ = 9.95 and *P*_*BH*_ <  0.0001), usual activities (ARI = 44%, odds_ordinal_ = 6.41 and *P*_*BH*_ <  0.0001), self-care (ARI = 35%, odds_ordinal_ = 6.96 and *P*_*BH*_ <  0.0001) and pain/discomfort (ARI = 37%, odds_ordinal_ = 3.12 and *P*_*BH*_ <  0.0001). Anxiety/depression was the less affected dimension in patients with renal insufficiency (ARI = 27%, odds_ordinal_ = 2.45 and *P*_*BH*_ < 0.001).

Diabetic patients showed values of ARI and odds_ordinal_ lower than those estimated for individuals with hypertension, renal insufficiency or back pain. The only significant values were obtained for anxiety/depression (ARI = 50%, odds_ordinal_ = 4.8 and *P*_*BH*_ < 0.0001) and mobility (ARI = 22%, odds_ordinal_ = 3.1 and *P*_*BH*_ = 0.0006) dimensions.

Similar results were obtained from the Improved RIDIT analysis of the EQ-5D-3 L data of the four diseases in comparison to the healthy group. These findings demonstrated the effectiveness of the Improved RIDIT method.

## Discussion

Researchers interested in understanding health related quality of life data adopt different strategies of analyzing data from the EQ-5D instrument. Most of these strategies are based on the use of the utility scores. The utility scores-based strategies provide information on the global health changes between two groups (ill/healthy, before/after treatment). Our findings suggest that using the Improved RIDIT approach provides a more detailed understanding of a health intervention or impact of pathology on quality of life outcomes for each dimension separately.

One example of EQ-5D analysis is to summarize frequencies corresponding to the responses obtained for each of the ordered severity levels. The chi-square test is often applied for statistical inference but does not take into consideration the ordinal nature of the severity of the questionnaire levels. The MWW test is an adequate test for ordinal data. Comparison between the Improved RIDIT and MWW tests using computer simulation showed that the improved RIDIT had less false positive rate than MWW for all sample sizes studied (Table 2). For sample sizes greater than 50, our simulation found that the two tests had similar power (Fig. 2). In addition, the MWW does not allow estimating the ARR/ARI and the NNT that the improved RIDIT provides.

Devlin et al. developed an approach based on the Pareto principle [14]. In this approach, an EQ-5D state of health is considered better than another if it is better in at least in one dimension and remains equivalent or better for the other dimensions. Conversely, a state of health is considered worse than another if it is worse at least in one dimension and it is not better in the remaining dimensions. On the basis of this principle to compare health states between two groups of patients, Devlin et al. found that following a hip replacement, 82% of patients had improved health, less than 5% had no change, less than 5% had worse health, and under 10% had a ‘mixed’ change [14].

Precise results were obtained when we applied the Improved RIDIT analyses on Devlin et al.’s data [14]. Devlin et al.’s approach makes it possible to evaluate the benefit of the treatment by considering the state of health in all five dimensions at the same time. The Improved RIDIT analysis allowed us to analyze the 5 dimensions separately, which makes it possible to analyze the effect of the treatment on each of the five dimensions. Our analyses revealed that the treatment resulted in significant improvement in pain/discomfort, anxiety/depression and usual activities. Understanding a specific impact of a treatment helps clinical teams better articulate potential quality of life outcomes to patients.

The Improved RIDIT statistic allows estimating the ARR/ARI and odds_Ordinal_, which are not estimated by the Pareto approach used by Devlin et al. Calculations using Pareto are based on the comparisons of the health status. When the EQ-5D-3 L is used there are 243 × 243 possible comparisons. This number is larger with the EQ-5D-5 L system (3125 × 3125). Pareto allows information about the global health and the improved RIDIT allows more information per dimension. The two approaches are complementary and can be used synergistically to analyze the EQ-5D data for evaluating the impact of treatment or disease on health outcomes.

By applying the Improved RIDIT method on diabetic Moroccan patients and the diabetic sample from Mateo’s study [23], we found similar findings. Diabetic Moroccan women were in a worse health state than diabetic men. The improved RIDIT added the information that significant differences between Moroccan diabetic women and men were observed for pain/discomfort (ARI = 0.33, odds_Ordinal_ = 3.06 and) and depression/anxiety (ARI = 0.43 and odds_Ordinal_ = 3.38) dimensions.

We also studied the relationship between the ordinal odds ratio and the absolute risk reduction/the absolute risk increase (ARR/ARI). The different analyzes of the EQ-5D data showed that in some cases the values of odds_Ordinal_ were high compared to their corresponding ARR/ARI. This can be explained, in these cases, by the small values of *π*^−^ (Fig. 3). We suggest to use only the ARR when π^−^ < 0.1, otherwise both ARR and odds_ordinal_ can be used to evaluate the impact of treatment or disease on the health-related quality of life.

Analyzing the EQ-5D data by the improved RIDIT allowed also estimating the NNT. For example, application of the approach on the data of Devlin [14], showed that the ARR of the Enzalutamide at week 13 was 7.5% (Table 4) for the anxiety dimension. It means that one have to treat NNT = 13 patients to prevent one additional bad anxiety outcome.

**Fig. 4:**
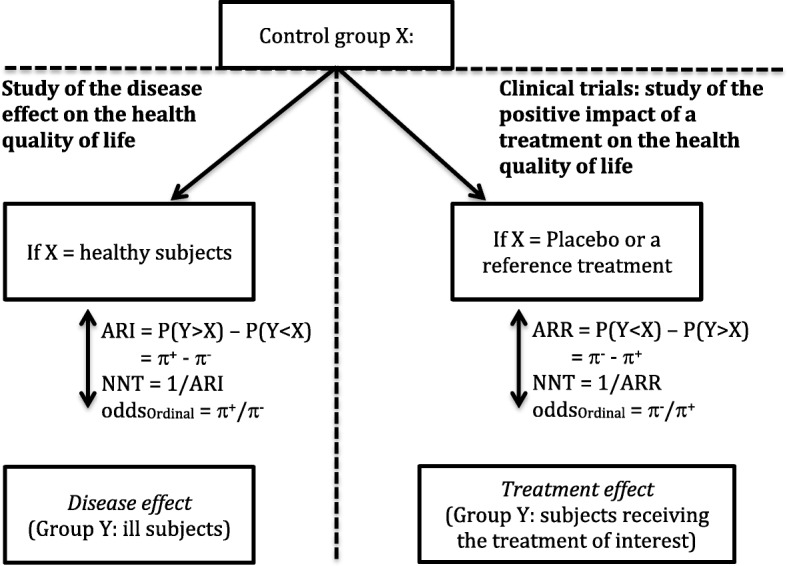
A Scheme for evaluating the effect of a disease or the effect of a treatment on the health-related quality of life by comparison of two randomly independent samples using the Improved RIDIT statistics. π + is the probability to be in severe health state and π- is the probability to be in a good health state. ARI: Absolute Risk Increase, ARR: Absolute Risk Reduction and NNT: Number Needed To Treat

To summarize all the information provided by the improved RIDIT method, we suggest using this statistic procedure to conduct analyses of the EQ-5D health-related quality of life outcomes from two randomly independent samples following the scheme in Fig. 4. The improved RIDIT method showed advantages of its use in the case of EQ-5D data obtained from two independent random samples by (i) calculating the W-test and taking into account for multiple testing (ii) estimating the ARR/ARI and the associated NNT and (iii) estimating the ordinal odds. It is of interest to extend the improved RIDIT approach to paired samples and for more than two samples.

The ARR/ARI, NNT and ordinal odds estimation are not allowed by either the MWW test or the utility-based approaches. Providing depth analysis of the EQ-5D data, the Improved RIDIT can be used to complete the information derived from utility scores.

## Conclusion

We utilized the Improved RIDIT statistic to examine the results of the EQ-5D surveys in two previously published studies and a data set we collected from Moroccans with chronic health conditions compared to a group of healthy individuals. This statistic procedure takes into account the severity level, which permits to estimate the absolute risk reduction/absolute risk increase and the ordinal odds ratio. The improved RIDIT analysis permits us to analyze the five dimensions of the EQ-5D separately, which gives clinical teams more precision in understanding the effect of a pathology or treatment on the health status.

## Supplementary information

**Additional file 1 Table S1.** Sample response frequencies (%) across 3 and 5 levels of EQ-5D dimensions from health-related quality of life outcomes of healthy subjects and sick subjects

**Additional file 2.**

## Data Availability

All data generated or analyzed during this study are included in this article.

## Abbreviations

ARI: Absolute Risk Increase
ARR: Absolute Risk Reduction
MWW: Mann-Whitney-Wilcoxon
NNT: Number Needed To Treat
