# Supplementary material for: Improved RIDIT statistic approach provides more intuitive and informative interpretation of EQ-5D data
Source: Health Qual Life Outcomes. 2020 Aug 21;18:63. doi: 10.1186/s12955-020-01313-3 (PMC7477837; doi:10.1186/s12955-020-01313-3)
Supplement: Supplementary file 1 — Additional file 1 Table S1. Sample response frequencies (%) across 3 and 5 levels of EQ-5D dimensions from health-related quality of life outcomes of healthy subjects and sick subjects [file 12955_2020_1313_MOESM1_ESM.docx]

**Table S1.** Sample response frequencies (%) across 3 and 5 levels of EQ-5D dimensions from health-related quality of life outcomes of healthy subjects and sick subjects

|  | **Control** | | | | **Hypertension** | | | | **Back Pain** | | | | **Diabetes** | | | | **Renal Insufficiency** | | | |
| --- | --- | --- | --- | --- | --- | --- | --- | --- | --- | --- | --- | --- | --- | --- | --- | --- | --- | --- | --- | --- |
| **Mobility** | **5L** |  | **3L** |  | **5L** |  | **3L** |  | **5L** |  | **3L** |  | **5L** |  | **3L** |  | **5L** |  | **3L** |  |
|  | 1 | 154 (0.86) | 1 | 154 (0.86) | 1 | 18 (0.23) | 1 | 18 (0.23) | 1 | 92 (0.58) | 1 | 92 (0.58) | 1 | 53 (0.65) | 1 | 53 (0.65) | 1 | 17 (0.3) | 1 | 17 (0.3) |
|  | 2 | 16 (0.09) |  |  | 2 | 30 (0.38) |  |  | 2 | 45 (0.28) |  |  | 2 | 13 (0.16) |  |  | 2 | 20 (0.36) |  |  |
|  | 3 | 9 (0.05) | 2 | 26 (0.14) | 3 | 27 (0.34) | 2 | 62 (0.78) | 3 | 17 (0.11) | 2 | 65 (0.41) | 3 | 16 (0.2) | 2 | 29 (0.35) | 3 | 5 (0.09) | 2 | 36 (0.64) |
|  | 4 | 1 (0.01) |  |  | 4 | 5 (0.06) |  |  | 4 | 3 (0.02) |  |  | 4 | 0 (0) |  |  | 4 | 11 (0.2) |  |  |
|  | 5 | 0 (0) | 3 | 0 (0) | 5 | 0 (0) | 3 | 0 (0) | 5 | 1 (0.01) | 3 | 1 (0.01) | 5 | 0 (0) | 3 | 0 (0) | 5 | 3 (0.05) | 3 | 3 (0.05) |
|  | **Total** | **180** |  | **180** |  | **80** |  | **80** |  | **158** |  | **158** |  | **82** |  | **82** |  | **56** |  | **56** |
| **Self**  **Care** | **5L** |  | **3L** |  | **5L** |  | **3L** |  | **5L** |  | **3L** |  | **5L** |  | **3L** |  | **5L** |  | **3L** |  |
|  | 1 | 173 (0.96) | 1 | 173 (0.96) | 1 | 50 (0.63) | 1 | 51 (0.64) | 1 | 109 (0.69) | 1 | 109 (0.69) | 1 | 75 (0.91) | 1 | 75 (0.91) | 1 | 35 (0.63) | 1 | 35 (0.63) |
|  | 2 | 5 (0.03) |  |  | 2 | 9 (0.11) |  |  | 2 | 28 (0.18) |  |  | 2 | 6 (0.07) |  |  | 2 | 4 (0.07) |  |  |
|  | 3 | 2 (0.01) | 2 | 7 (0.04) | 3 | 16 (0.2) | 2 | 28 (0.35) | 3 | 19 (0.12) | 2 | 48 (0.3) | 3 | 1 (0.01) | 2 | 7 (0.09) | 3 | 6 (0.11) | 2 | 12 (0.21) |
|  | 4 | 0 (0) |  |  | 4 | 4 (0.05) |  |  | 4 | 1 (0.01) |  |  | 4 | 0 (0) |  |  | 4 | 2 (0.04) |  |  |
|  | 5 | 0 (0) | 3 | 0 (0) | 5 | 1 (0.01) | 3 | 1 (0.01) | 5 | 1 (0.01) | 3 | 1 (0.01) | 5 | 0 (0) | 3 | 0 (0) | 5 | 9 (0.16) | 3 | 9 (0.16) |
|  | **Total** | **180** |  | **180** |  | **80** |  | **80** |  | **158** |  | **158** |  | **82** |  | **82** |  | **56** |  | **56** |
| **Usual**  **Activities** | **5L** |  | **3L** |  | **5L** |  | **3L** |  | **5L** |  | **3L** |  | **5L** |  | **3L** |  | **5L** |  | **3L** |  |
|  | 1 | 151 (0.84) | 1 | 151 (0.84) | 1 | 43 (0.54) | 1 | 43 (0.54) | 1 | 86 (0.54) | 1 | 86 (0.54) | 1 | 64 (0.78) | 1 | 64 (0.78) | 1 | 24 (0.43) | 1 | 24 (0.43) |
|  | 2 | 20 (0.11) |  |  | 2 | 18 (0.23) |  |  | 2 | 30 (0.19) |  |  | 2 | 10 (0.12) |  |  | 2 | 14 (0.25) |  |  |
|  | 3 | 8 (0.04) | 2 | 29 (0.16) | 3 | 12 (0.15) | 2 | 35 (0.44) | 3 | 30 (0.19) | 2 | 68 (0.43) | 3 | 7 (0.09) | 2 | 17 (0.21) | 3 | 5 (0.09) | 2 | 24 (0.43) |
|  | 4 | 1 (0.01) |  |  | 4 | 6 (0.08) |  |  | 4 | 8 (0.05) |  |  | 4 | 0 (0) |  |  | 4 | 5 (0.09) |  |  |
|  | 5 | 0 (0) | 3 | 0 (0) | 5 | 1 (0.01) | 3 | 2 (0.03) | 5 | 4 (0.03) | 3 | 4 (0.03) | 5 | 1 (0.01) | 3 | 1 (0.01) | 5 | 8 (0.14) | 3 | 8 (0.14) |
|  | **Total** | **180** |  | **180** |  | **80** |  | **80** |  | **158** |  | **158** |  | **82** |  | **82** |  | **56** |  | **56** |
| **Pain/**  **Discomfort** | **5L** |  | **3L** |  | **5L** |  | **3L** |  | **5L** |  | **3L** |  | **5L** |  | **3L** |  | **5L** |  | **3L** |  |
|  | 1 | 99 (0.55) | 1 | 99 (0.55) | 1 | 17 (0.21) | 1 | 17 (0.21) | 1 | 0 (0) | 1 | 0 (0) | 1 | 44 (0.54) | 1 | 44 (0.54) | 1 | 16 (0.29) | 1 | 16 (0.29) |
|  | 2 | 55 (0.31) |  |  | 2 | 16 (0.2) |  |  | 2 | 63 (0.4) |  |  | 2 | 10 (0.12) |  |  | 2 | 18 (0.32) |  |  |
|  | 3 | 24 (0.13) | 2 | 81 (0.45) | 3 | 43 (0.54) | 2 | 59 (0.74) | 3 | 69 (0.44) | 2 | 152 (0.96) | 3 | 19 (0.23) | 2 | 29 (0.35) | 3 | 7 (0.13) | 2 | 31 (0.55) |
|  | 4 | 2 (0.01) |  |  | 4 | 0 (0) |  |  | 4 | 20 (0.13) |  |  | 4 | 0 (0) |  |  | 4 | 6 (0.11) |  |  |
|  | 5 | 0 (0) | 3 | 0 (0) | 5 | 4 (0.05) | 3 | 4 (0.05) | 5 | 6 (0.04) | 3 | 6 (0.04) | 5 | 9 (0.11) | 3 | 9 (0.11) | 5 | 9 (0.16) | 3 | 9 (0.16) |
|  | **Total** | **180** |  | **180** |  | **80** |  | **80** |  | **158** |  | **158** |  | **82** |  | **82** |  | **56** |  | **56** |
| **Anxiety/**  **Depression** | **5L** |  | **3L** |  | **5L** |  | **3L** |  | **5L** |  | **3L** |  | **5L** |  | **3L** |  | **5L** |  | **3L** |  |
|  | 1 | 107 (0.59) | 1 | 107 (0.59) | 1 | 14 (0.18) | 1 | 15 (0.19) | 1 | 45 (0.28) | 1 | 45 (0.28) | 1 | 21 (0.26) | 1 | 21 (0.26) | 1 | 21 (0.38) | 1 | 21 (0.38) |
|  | 2 | 57 (0.32) |  |  | 2 | 5 (0.06) |  |  | 2 | 70 (0.44) |  |  | 2 | 17 (0.21) |  |  | 2 | 22 (0.39) |  |  |
|  | 3 | 11 (0.06) | 2 | 73 (0.41) | 3 | 42 (0.53) | 2 | 46 (0.58) | 3 | 30 (0.19) | 2 | 112 (0.71) | 3 | 27 (0.33) | 2 | 44 (0.54) | 3 | 1 (0.02) | 2 | 31 (0.55) |
|  | 4 | 5 (0.03) |  |  | 4 | 0 (0) |  |  | 4 | 12 (0.08) |  |  | 4 | 0 (0) |  |  | 4 | 8 (0.14) |  |  |
|  | 5 | 0 (0) | 3 | 0 (0) | 5 | 19 (0.24) | 3 | 19 (0.24) | 5 | 1 (0.01) | 3 | 1 (0.01) | 5 | 17 (0.21) | 3 | 17 (0.21) | 5 | 4 (0.07) | 3 | 4 (0.07) |
|  | **Total** | **180** |  | **180** |  | **80** |  | **80** |  | **158** |  | **158** |  | **82** |  | **82** |  | **56** |  | **56** |
